# Transposable elements in normal and malignant hematopoiesis

**DOI:** 10.1242/dmm.050170

**Published:** 2023-07-28

**Authors:** Eline Lemerle, Eirini Trompouki

**Affiliations:** IRCAN Institute for Research on Cancer and Aging, INSERM Unité 1081, CNRS UMR 7284, Université Côte d'Azur, Nice, France

## Abstract

Transposable elements (TEs) are dispersed repetitive DNA sequences that can move within a genome. Even though hundreds of years of evolution have led to the accumulation of mutations that render most TEs unable to transpose, they still exert multiple important functions. They play a role in hematopoiesis, especially during periods of high cellular plasticity, such as development, regeneration and aging. This is because TEs can populate functional elements, such as enhancers. Furthermore, TE RNA can be sensed by innate immune sensors that play a role in inflammation and inflammaging. TEs also play an important role in different aspects of leukemia and lymphoma, leading to either beneficial or detrimental outcomes. Further studies into the function of TEs in healthy or diseased hematopoietic systems are necessary to manipulate them for therapeutic benefit.

## Teaser

**Summary:** Transposable elements exert a functional role on hematopoiesis and disease throughout life. Further characterization of these repetitive elements will open new diagnostic and therapeutic avenues.

## Introduction

Transposable elements (TEs) – also known as transposons, interspersed repeats, selfish genetic elements, jumping genes or parasitic DNA – are dispersed repetitive sequences that can move within a genome by a mechanism called transposition. They were discovered in the 1940s in maize by Barbara McClintock (McClintock, 1950). McClintock's results, however, were considered controversial until the discovery of TEs in bacteria (Shapiro, 1969) and *Drosophila* (Kidwell et al., 1977). Since then, genomic sequencing has led to the recognition of TEs as main components of eukaryotic genomes (Huang et al., 2012), making up ∼45% of the human genome (Lander et al., 2001).

TEs are grouped into two main classes according to their mode of transposition (Finnegan, 1989; Wicker et al., 2007). Class 1 TEs – also known as retrotransposons – transpose into the genome by a ‘copy-and-paste’ mechanism during which an RNA intermediate is reverse-transcribed into a cDNA copy (Bourque et al., 2018). These retrotransposons can be further divided into five subclasses based on their organization, mechanistic features and reverse transcriptase phylogeny, i.e. (1) long-terminal repeat (LTR) retrotransposons (Box 1), (2) *Dictyostelium* intermediate repeat sequence 1 (DIRS-1)-like elements (Box 1), (3) *Penelope*-like elements (PLEs; Box 1), (4) long interspersed nuclear elements (LINEs) and (5) short interspersed nuclear elements (SINEs) (Box 1). Class 2 TEs are known as DNA or ‘cut-and-paste’ transposons (Box 1) (Greenblatt and Alexander Brink, 1963). They are divided into two subclasses according to the number of DNA strands that are cut during transposition. Subclass I TEs transpose into the genome mostly by a ‘cut-and-paste’ mechanism via a DNA intermediate; they are characterized by their terminal inverted repeats (TIRs) of variable length. Subclass 2 TEs, including helitrons (Box 1) and mavericks (Box 1), undergo transposition via replication without double-stranded cleavage.

**Box 1. Glossary**

**Acute lymphoblastic leukemia (ALL):** a type of cancer in which the bone marrow makes too many lymphocytes.

**Basic helix-loop-helix ARNT-like (BMAL1):** a protein that, in humans, is encoded by the *BMAL1* gene on chromosome 11. It plays a key role as a positive component of the mammalian autoregulatory transcription-translation feedback loop (TTFL), a  negative feedback loop that is responsible for generating molecular circadian rhythms. *BMAL1* is the only essential clock gene for the circadian rhythm in humans.

**Breakage−fusion–bridge cycle:** genetic instability in which the end-to-end fusion of broken sister chromatids yields a dicentric chromosome that forms a bridge in mitosis during anaphase.

**cGAS–STING (cyclic GMP–AMP synthase-stimulator of interferon genes) pathway:** a key component of the innate immune system that functions to detect the presence of cytosolic DNA, triggering the expression of inflammatory genes that can lead to senescence or activation of cell-autonomous defense mechanisms.

**Deletion:** genomic instability in which part of a chromosome or DNA sequence is omitted during DNA replication.

***Dictyostelium* intermediate repeat sequence 1 (DIRS-1)-like elements:** members of the non-LTR retrotransposon subclass. They use a tyrosine recombinase instead of an integrase to integrate into the genome.

**DNA methylation:** a process predominantly involving the nucleotide cytosine during which a methyl group is transferred onto its C5 position, yielding 5-methylcytosine. This mostly acts to suppress gene transcription and repress TEs.

**DNA methyltransferase (DNMT) inhibitors:** drugs used to study the role of DNA methylation in different tissues and model systems. They represent a possible therapeutic option for disorders that involve altered DNA methylation.

**DNA strands:** polymers or chains of the monomer deoxyadenosine monophosphate (dAMP) that are linked by phosphodiester bonds.

**DNA transposons:** DNA sequences that can move to and integrate at different locations within the genome.

**Duplication (also known as amplification):** genomic instability in which new genetic material is generated during molecular evolution. It can be defined as any duplication of a region of DNA that contains a gene.

**Endogenous retroviruses (ERVs):** endogenous viral elements of the genome that closely resemble and might be derived from retroviruses. Human ERVs (HERVs) are a type of LTR retrotransposon and account for 5–8% of the human genome.

**Endothelial-to-hematopoietic transition:** a highly plastic process during which an endothelial cell becomes a hemogenic endothelial cell from which HSPCs will emerge.

**Evolutionarily young TEs:** a subset of TEs that have more recently transposed into the human germline (Ohtani et al., 2018, 2020).

**H3K9me3:** an epigenetic modification to the DNA packaging protein histone H3. It indicates histone H3 tri-methylated at lysine residue 9 and is often associated with heterochromatin.

**Histone modifications:** acetylation, methylation, phosphorylation and ubiquitylation are the main modifications of histones, regulating chromatin structure and transcriptional activity (Bannister and Kouzarides, 2011).

**Helitrons:** eukaryotic rolling-circle transposable elements that are proposed to transpose by a rolling circle replication mechanism via a single-stranded DNA intermediate (Grabundzija et al., 2016).

**Heterochromatin:** a ‘closed’ conformation of the chromatin-decreased transcription due to restricted access of transcription factors to DNA.

**Heterochromatin decondensation:** an ‘open’ conformation of chromatin due to the decreased binding affinity between histones and DNA. This allows transcription factors to access DNA, resulting in transcription activation.

**HERVs:** see ‘Endogenous retroviruses (ERVs)’.

**Human silencing hub (HUSH) complex:** a novel epigenetic complex responsible for the variability of the positional effect of integrated transgenes in human cells. It is associated with genomic regions dense in H3K9me3.

**Hutchinson–Gilford progeria syndrome (HGPS):** a rare genetic disorder caused by a mutation in the LAMIN A gene *LMNA* that leads to accelerated aging.

**Leukemic stem cells (LSCs) in acute myeloid leukemia (AML):** a low-frequency subpopulation of leukemic cells that possess stem cell properties distinct from those of the majority of leukemic cells, including the ability of self-renewal and drug resistance.

**Long interspersed nuclear elements (LINEs):** a subclass of ∼7000 bp-long non-LTR retrotransposons that are widespread in the genomes of many eukaryotes (21.1% of human genome). The most abundant LINE in humans is LINE-1. Their integration into the genome is coupled to a process called target-primed reverse transcription (Luan et al., 1993).

**Long-terminal repeats (LTRs):** a pair of identical DNA sequences that are several hundred base pairs long and flank a retrotransposon. LTRs occur in eukaryotic genomes and flank gene or pseudogene series; they contain signals that promote and terminate transcription and are required for reverse transcription processes.

**LTR retrotransposon:** class I transposable elements, i.e. retrotransposons, characterized by the presence of LTRs directly flanking an internal coding region. Their chromosomal integration relies on cleavage and strand-transfer reactions catalyzed by integrase (Brown et al., 1987). This includes human endogenous retrovirus (ERV) elements.

**Mavericks (also known as polintons):** transposons that undergo replicative transposition without RNA intermediates (Kapitonov and Jurka, 2006).

**Mosaicism:** the coexistence of two or more cell populations of different genotype in one individual organism.

**M-phase phosphoprotein 8 (MPHOSPH8, also known as MPP8):** a protein component of the human silencing hub (HUSH) complex that specifically binds H3K9me3 histone H3. It is vital for the formation of heterochromatin and has specific roles in cancer metastasis.

**Mutagenesis:** a process by which the DNA of an organism is permanently changed and a gene mutation occurs. It can be natural or artificial, and can cause cancer, hereditary diseases or evolutionary innovations. It is the main cause of species biodiversity.

**Non-LTR retrotransposon:** class I transposable elements, i.e. retrotransposons, characterized by the lack of LTRs. cDNA synthesis does not take place in the cytoplasm of the cell but at the site of insertion of the new copy.

**Pancreatic ductal adenocarcinoma (PDAC):** the most-prevalent type of pancreatic neoplasm, developed in the exocrine compartment and accounting for >90% of pancreatic cancer cases.

***Penelope*-like elements (PLEs):** a subclass of eukaryotic retroelements characterized by a reverse transcriptase domain with similarity to telomerases and group II introns.

**R/G ratio (TE repeat transcript to gene transcript ratio):** ratio between the median of the normalized counts for all TE transcripts relative to that of the normalized counts for all gene transcripts.

**Retinoic acid-inducible gene I (RIG-I)-like receptors (RLRs):** a type of intracellular pattern recognition receptor involved in viral recognition by the innate immune system. RIG-I (retinoic-acid inducible gene) is the best characterized receptor of the RLR family. Together with MDA5 (melanoma differentiation-associated 5) and LGP2 (laboratory of genetics and physiology 2), they are sentinels for intracellular viral RNA, the product of viral infection.

**Retrotransposon:** mobile genetic element amplified by reverse transcription via an RNA intermediate, which can then move to and integrate at – i.e. transpose to – different locations within the genome. Copy numbers of a retrotransposon are, therefore, increased quicker than DNA transposons.

**SET domain bifurcated histone lysine methyltransferase 1 (SETDB1):** a prominent member of the Suppressor of Variegation 3–9 (SUV39)-related protein lysine methyltransferases (PKMTs). It is widely expressed in human tissues, methylating Histone 3 lysine 9 (H3K9) residues, promoting chromatin compaction and exerting negative regulation on gene expression (Markouli et al., 2021).

**Short dispersed nuclear elements (SINEs):** a subclass of retrotransposons that are ∼100-700 bp. They make up about 13% of the mammalian genome. Their integration is also coupled to target-primed reverse transcription (Luan et al., 1993).

**Sirtuin 6 (SIRT6):** a histone deacetylase of the sirtuin family expressed in the nucleus. It is involved in DNA repair, telomere maintenance, carbohydrate and lipid metabolism, and plays a protective role against senescence.

**Sirtuin 7 (SIRT7):** a NAD-dependent deacetylase that interacts with RNA polymerase I and has a key role in chromatin remodeling.

**Target-primed reverse transcription:** a process in which an element-encoded endonuclease (an endonuclease that is encoded directly by the transposable element) cuts the target DNA, generating an exposed 3′ hydroxyl group that serves as a primer for reverse transcription of the RNA of that element.

**Translocation:** genomic instability characterized by the reciprocal exchange of chromosomal material between non-homologous chromosomes, i.e. those that do not belong to the same pair.

**Werner syndrome (WS):** a rare autosomal recessive disorder with mutations in the *WRN* gene. It is characterized by the appearance of premature aging and is also known as adult progeria.

**Fig. 1.**
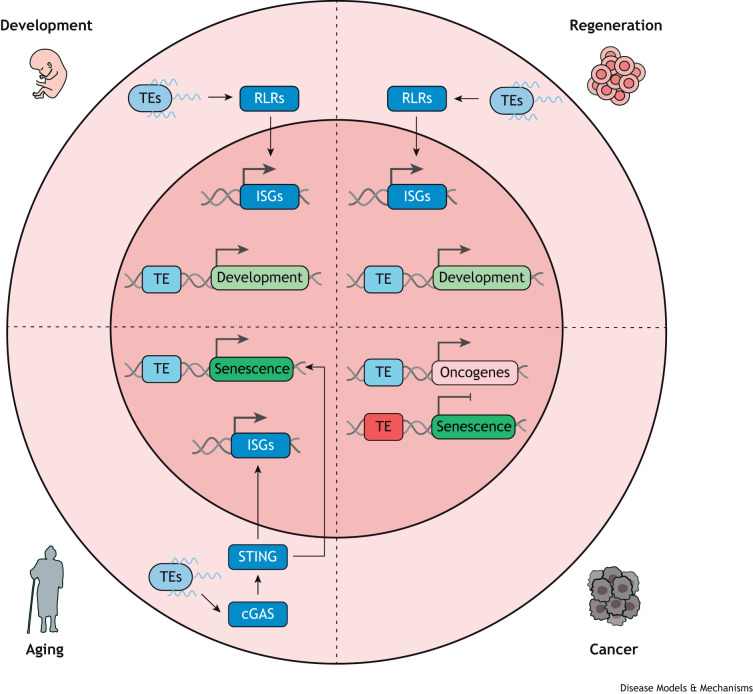
**During development TEs can act as enhancers of developmental genes and, through their transcription, they can act as ligands for RNA sensors, such as RLRs, which then activate ISGs to induce a beneficial inflammatory response that drives HSPC formation.** During regeneration, TEs perform, again, as enhancers of developmental genes, and activate the RLR-mediated inflammatory response allowing cells to exit quiescence. During aging, transcribed TEs can activate the immune and inflammatory response through the cGAS–STING pathway leading to senescence, and can more directly induce the transcription of senescence genes as enhancers. In the context of cancer, TEs can act as enhancers of oncogenes by a mechanism called onco-exaptation, and as repressors of senescence genes to allow cell proliferation. cGAS–STING, cyclic GMP–AMP synthase-stimulator of interferon genes; HSPC, hematopoietic stem and progenitor cell; ISG, interferon-stimulated gene; RLR, retinoic acid-inducible gene-like receptor; TE, transposable element.

Long considered as junk DNA, TEs are now known to have many important functions. They have been important factors in evolution because of their ability to generate genetic diversity, which involves the remodeling of processes in the eukaryotic genome (Zattera and Bruschi, 2022) and the introduction of mutations (Gagnier et al., 2019). Expression of TEs is controlled through several mechanisms that involve small RNA, chromatin modifications, DNA modification pathways (Berrens et al., 2017; Goodier, 2016; Liu et al., 2018; Molaro and Malik, 2016) and repressors, such as the Krüppel associated box (KRAB) domain-containing zinc-finger proteins (Ecco et al., 2017; Imbeault and Trono, 2014; Imbeault et al., 2017; Yang et al., 2017). TEs are mainly silent (Cogoni and Macino, 2000; Girard and Hannon, 2008) owing to epigenetic modifications – such as DNA methylation (Box 1) that suppresses methylated gene expression (Zardo, 2021), and histone modifications (Box 1) that regulate chromatin structure and, therefore, gene transcription – together with other mechanisms (Choi and Lee, 2020). For example, TRIM28 (also known as KAP1) recruits the ‘histone H3 at lysine 9 (H3K9)’ methyltransferase SETDB1 and CBX1 (also known as HP1) to form heterochromatin (Box 1) at TE sequences to repress their expression (Schultz et al., 2002). However, it is now known that TEs exert a strong influence on host biology through their de-repression and activation (Horváth et al., 2017) following various stress signals. For example, they can affect gene expression (Chuong et al., 2017; Fueyo et al., 2022; Tsai et al., 2022), tissue regeneration (Angileri et al., 2022) and adaptive immunity (Hayward and Gilbert, 2022) (Fig. 1). Moreover, precise regulation of TE activation is crucial for internal gestation (Lu et al., 2017), mammalian embryonic genome activation (Li et al., 2022) and normal embryonic development and organogenesis. Furthermore, there is increasing evidence for the role of TEs in diseases like leukemia, and immune and neurological diseases, among others (Bourque et al., 2018; Gorbunova et al., 2021).

Synthetic TEs, like *Sleeping beauty* and *PiggyBac*, have been developed as new molecular tools for gene therapy that enable stable gene transfer and sustained transgene expression (Kebriaei et al., 2017). TE-derived elements can also be used in expression vectors to improve transcription regulation of the genes they contain (Palazzo and Marsano, 2021), can be harnessed in functional genomics and, potentially, in TE-based forward and reverse mutagenesis screens (Kawakami et al., 2017).

Among the different systems they affect, TEs have been shown to exert roles – both beneficial (physiological) and detrimental (pathological) – during hematopoietic development, regeneration, aging and disease.

Owing to the many diverse functions of TEs, it is important to understand their function, especially since they have been understudied for many years. Among the different systems they affect, TEs have been shown to exert roles – both beneficial (physiological) and detrimental (pathological) – during hematopoietic development, regeneration, aging and disease. These roles are the focus of this Perspective.

## The role of TEs in hematopoiesis

Hematopoietic stem and progenitor cells (HSPCs) are formed in vertebrates by an endothelial-to-hematopoietic transition (Box 1), which is a highly plastic process (Ottersbach, 2019). During this process, intrinsic and extrinsic signaling pathways are activated or inhibited, which is necessary for blood formation. Several studies have highlighted the importance of inflammation in regulating the development of embryonic HSPCs (Espín-Palazón et al., 2014; He et al., 2015; Li et al., 2014; Sawamiphak et al., 2014; Zhong et al., 2023). In zebrafish, various innate immune sensors, including retinoic acid-inducible gene I (RIG-I)-like receptors (RLRs; Box 1), have been implicated in the emergence of HSPCs (Lefkopoulos et al., 2020). The study by Lefkopoulos and colleagues showed that different TEs are transcribed in endothelial, hemogenic cells and in HSPCs. These TEs then triggered expression of RLRs, including that of *RIG-I* (also known as *rigi*) and *melanoma differentiation-associated gene 5* (*mda5*, also known as *ifih1*), which activated inflammatory signaling to enhance the formation of HSPCs. This suggests that, during a plastic process, TE expression increases and – until their repression – that these TEs are used as ligands by RLRs to ‘sense’ plasticity. In this scenario, moderate and tolerable plastic changes result in beneficial inflammatory signaling that drives HSPC formation; however, extreme changes may lead to inflammation that kills the cells.

Hematopoietic stem cells (HSCs) in adult bone marrow (BM) are present in a quiescent state, and, under homeostatic conditions, human HSCs exhibit very low transcription and transposition levels of LINE-1 subclass retrotransposons (Kazachenka et al., 2019; Schumann et al., 2019) [see Glossary ‘Long interspersed nuclear elements (LINEs)’, Box 1]. However, when stress occurs, HSCs exit their quiescent state, and divide and reconstitute the BM in a highly plastic process that simulates the aforementioned formation of HSPCs. This rapid adaptability of HSCs partly relies on epigenetic and epitranscriptomic regulators that affect the silencing, expression and stability of TEs (Clapes and Trompouki, 2020). Overall, there is variable expression of TEs during the development of HSPCs, whereas – in the highly quiescent HSCs – expression of TEs is considerably lower. Under stress conditions, such as ionizing radiation, expression and transposition of LINE-1 retrotransposons increases in mouse HSCs, leading to persistent accumulation of phosphorylated H2A histone family member X (H2AX) foci, a marker of DNA damage and loss of function of HSCs (Barbieri et al., 2018). Moreover, upon irradiation, reduced NF-κB signaling leads to a loss of H3K9me3 (Box 1) in intronic LINE-1 in mouse HSCs (Pelinski et al., 2022). This de-repression of LINE-1 results in the inhibition of HSC-specific gene expression. Indeed, pre-treatment with TNFα can rescue all irradiation-associated defects in mouse HSCs (Pelinski et al., 2022) and, interestingly, the activity of LINE-1 retrotransposons can also be restrained by thrombopoietic-mediated interferon induction (Barbieri et al., 2018). Thus, induction of NF-κB or interferon signaling can maintain HSCs exposed to stress by moderating TE activation. The involvement of TEs in hematopoietic regeneration after chemotherapy has also been established in mice (Clapes et al., 2021), i.e. after chemotherapy, there was increased transcription of TEs that bind to and activate the innate immune receptor MDA5 in mice, thereby generating an inflammatory response necessary for the HSCs to exit quiescence. *Mda5*^−/−^ HSCs were more quiescent compared with WT HSCs, therefore performing better in the long term. However, during rapid acute stress, such as serial injections of chemotherapeutic fluorouracil, *Mda5*^−/−^ mice were unable to reconstitute their blood system fast enough (Clapes et al., 2021). These varying studies demonstrate the importance of fine-tuning TE regulation in hematopoietic functions.

Natural stress, such as aging, is also linked to alterations in the transcription of TEs (Cecco et al., 2019; Gorbunova et al., 2021; Liu et al., 2023; Simon et al., 2019). Aging leads to upregulation of various TEs that are usually silent in HSCs of young mice (Sun et al., 2014). However, most of the studies investigating aging and TEs are done in non-hematopoietic tissues. Liu and colleagues showed de-repression of a TE within the family of human endogenous retroviruses (HERVs; Box 1) – i.e. HERV-K (with K denoting a lysine tRNA primer-binding site) – in senescent human muscle precursor cells within two models of premature aging, namely Hutchinson-Gilford progeria syndrome (HGPS) and Werner syndrome (WS) (Liu et al., 2023). The group found that HERV-K induced the production of retrovirus-like particles (RVLPs) that can elicit senescence and mediate age-promoting effects in young cells. Therefore, repression of HERV-K might alleviate senescence (Liu et al., 2023). A recent study has shown that deficiency of the basic helix-loop-helix ARNT-like (BMAL1) protein (Box 1) yields an accelerated aging phenotype, activating LINE-1 transcription in human and cynomolgus monkey MPCs, and accelerating senescence through the cGAS-STING signaling pathway (Box 1) (Liang et al., 2022). Similarly, depletion of sirtuin 6 (SIRT6) (Box 1) in human dermal fibroblast or mouse embryonic fibroblast cells in response to stress- and aging-induced DNA damage leads to the decondensation of heterochromatin (Box 1) and activation of LINE-1 transcription (Van Meter et al., 2014). Furthermore, deficiency in SIRT7 in aging human mesenchymal stem cells leads to loss of heterochromatin in LINE-1 loci, leading to its reactivation and, eventually, induction of innate immune signaling via the cGAS–STING pathway (Bi et al., 2020).

Collectively, TEs exert diverse roles in highly plastic biological processes, such as developmental transitions during normal physiological processes or in response to stress and aging.

## The role of TEs in hematological cancers

We know that almost half of the genome consists of TEs that are mostly inactive under homeostatic conditions in adults (Luqman-Fatah and Miyoshi, 2022). However, *de novo* retrotransposition can occur (Rodriguez-Martin et al., 2020; Tubio et al., 2014). A pan-cancer analysis of whole genomes has shown that an increase in LINE-1 expression drives genomic instability by promoting deletion, translocation, duplication and breakage–fusion–bridge cycles (Box 1) of genetic material, thus, leading to cancer (Rodriguez-Martin et al., 2020). Cancer cells have been reported to display LINE-1 dysregulation, leading to further mutagenesis and mosaicism (Box 1), which drives metastasis (Rodriguez-Martin et al., 2020).

Various HERV LTR retrotransposons are expressed in different hematological malignancies. For example, the HERV-E (with E denoting a glutamate tRNA primer-binding site) family is expressed in acute megakaryocytic and erythroid leukemia (Engel et al., 2021). HERVs are also important in various cases of lymphoma and leukemia (Stricker et al., 2023). In 1997, HERV LTR retrotransposons were identified to drive translocation of chromosome 14q32 to chromosome 7q21 in a female patient with B-cell chronic lymphocytic leukemia (B-cell CLL) (Wahbi et al., 1997), and other studies have followed. In a patient with atypical stem cell myeloproliferative disorder, *FGFR1* was found to be constitutively expressed due to a fusion with HERV-K3 (Guasch et al., 2003; Mugneret et al., 2000). Additionally, Leung et al. reported that infection of primary B lymphocytes and lymphoblastoid cell lines with Epstein-Barr virus leads to the activation of HERV LTR retrotransposons and, subsequently, enhances the activation of genes important for oncogenesis (Leung et al., 2018).

One type of leukemia that has been more extensively studied for its association with TE expression is acute myeloid leukemia (AML), a heterogeneous cancer of the myeloid lineage. Leukemic stem cells (LSCs; Box 1) propagate AML through their self-renewal capacity and by sustaining long-term maintenance of the malignant population (Eppert et al., 2011; Shlush et al., 2017). Interestingly, a significant proportion of TEs are overexpressed in AML during a process called onco-exaptation, defined as the epigenetic reactivation of TEs that act as cryptic promoters, driving oncogene expression in cancer (Babaian and Mager, 2016; Jang et al., 2019). For example, Deniz et al. have identified six ERV families with AML-associated enhancer chromatin signatures and, when these were deleted or silenced in leukemia cell lines, apoptosis occurred (Deniz et al., 2020). TEs can also be used to classify AML patients. Colombo et al. examined the expression of TEs in 178 adult AML patients. They found 14 TE transcripts that could be used to establish a prognosis in AML patients, such as high or low AML risk, and that are independent of gene mutations (Colombo et al., 2018). Along the same line, Onishi-Seebacher et al. demonstrated that the ratio of all TE repeats (R) to the overall gene (G) expression (R/G ratio; Box 1) could serve as a prognostic biomarker, with a high R/G ratio identifying a favorable prognosis and a low R/G ratio identifying a poor prognosis (Onishi-Seebacher et al., 2021).

[…] the different regulatory pathways of TEs need to be carefully studied and dissected to enable better management of cancer.

AML is often associated with epigenetic alterations in DNA methylation and modification of histones. In a 2021 preprint, Nadorp et al. revealed that LSCs and mature leukemic cells display different chromatin accessibility profiles for diverse TE subfamilies (Nadorp et al., 2021 preprint). Interestingly, TEs located in the accessible chromatin of LSCs act as docking sites for several oncogenic drivers of AML, including LYL1, a proto-oncogene of the TAL family (Nadorp et al., 2021). By contrast, a large proportion of the TE pool is repressed by methylation in AML (Capone et al., 2018). For example, silencing of LINE-1 is associated with poor prognosis in AML (Gu et al., 2021). Furthermore, the methyltransferase SETDB1 (Box 1) is overexpressed in many cancers, including AML (Cuellar et al., 2017). Interestingly, Cuellar et al. showed that knockdown of SETDB1 in AML cells triggers TE expression that leads to the production of double-stranded RNAs (dsRNAs) and increases interferon signaling and apoptosis (Cuellar et al., 2017). Along the same line, the use of DNA methyltransferase (DNMT) inhibitors (Box 1) in the treatment of hematologic cancers allows the activation of specific subsets of evolutionarily young TEs (Box 1), leading to the upregulation of tumor suppressor genes and to the activation of an inflammatory response (Greve et al., 2021; Ohtani et al., 2020). DNMT inhibitors or loss of M-phase phosphoprotein 8 (MPHOSPH8, also known as MPP8) – a member of the human silencing hub (HUSH) complex (Box 1) – can reactivate LINE-1, thereby inducing DNA damage response and cell cycle exit in leukemic cells (Gu et al., 2021). It is also interesting to note that TE expression mirrors the epigenetic traits of the cell of origin, meaning that TE expression can reveal the cell of origin, as shown in pancreatic ductal adenocarcinoma (Espinet et al., 2021).

In the context of cancer, including AML, TEs play a dual role (Colombo et al., 2018; Grillo and Lupien, 2022). On the one hand, transcription and activation of TEs contribute to cancer development due to their mobility, potentially leading to genomic instability and mutagenesis (Rodriguez-Martin et al., 2020), as well as their involvement in onco-exaptation, i.e. the recruitment of TE-derived promoters to drive expression of oncogenes and, subsequently, promote oncogenesis (Babaian and Mager, 2016; Jang et al., 2019). On the other hand, reactivation of TEs in hematopoietic and other types of cancer can be beneficial, as shown, for example, by inducing interferon signaling and cancer cell death (Chiappinelli et al., 2015; Colombo et al., 2018; Espinet et al., 2021; Ohtani et al., 2020). Therefore, the different regulatory pathways of TEs need to be carefully studied and dissected to enable better management of cancer.

## Future opportunities in TE research

The accumulation of genomic data over the past two decades has led to substantial progress in the field of TE research. Emerging genomics methods, including long-read and single-cell RNA sequencing, have the potential to revolutionize our understanding of TE biology and regulation. As the quality and quantity of genomics resources increase, many more instances of TE function in development and disease are likely to be discovered (Hayward and Gilbert, 2022).

[…] we must aim to better understand how HSC-responsive genes might be regulated by elements populated by TEs.

Current evidence shows that TEs are not only junk DNA but that they have multiple functions in all the roadblocks of life – from development to regeneration, to aging and disease. Indeed, as mentioned above, all these are phases in life during which cells exhibit high plasticity. Upon changes that lead to increased cellular plasticity TEs are expressed and have a role in innate immune signaling or as functional genomics elements. For the latter, evidence is emerging mostly from studies of development (Bourque et al., 2018; Senft and Macfarlan, 2021), but not of the hematopoietic system. Therefore, more research focusing on the role of TEs, specifically in hematopoiesis, is required.

Regarding aging, it has been shown in other systems – including adipose tissue, liver, kidney and muscle – that manipulating the biology of TEs, e.g. with reverse L1 transcriptase inhibitors, can reverse phenotypes of aging in mice (Cecco et al., 2019). Therefore, research focusing on the hematopoietic system in this context could aid its rejuvenation. Indeed, we must aim to better understand how HSC-responsive genes might be regulated by elements populated by TEs. Additionally, we have to understand the mechanism of TE upregulation upon stress, since such studies in the hematopoietic system are currently limited. To accelerate this, molecular biology studies that show, for example, integration of TEs into the genome (Sultana et al., 2019), could be done in cell systems and might reveal regulatory roles of TEs.

Further studies that increase our understanding of the role of TEs in different systems will substantially contribute to our knowledge of the biology behind normal physiology and pathogenesis and may also reveal novel therapeutics

Advancing our knowledge of TEs in cancer should allow a more personalized treatment approach. In the case of AML, treatment could start with analyzing deregulated TEs in LSCs obtained through biopsies, followed by assessing of whether TE inhibition or TE re-expression is needed for therapy. Immuno- or chemotherapy could then be combined with treatment targeting regulators of TE expression to force LSC differentiation into mature leukemic cells and to reactivate immune signaling pathways leading to cell death (Capone et al., 2018). Such methods could be broadly applicable, which renders the investigation of the functions of TEs in several types of leukemia a promising therapeutic avenue. To this end the methytransferase inhibitors decitabine and azacytidine are already used as combination therapies to treat AML but also other types of leukemia (Cheung et al., 2023; DiNardo et al., 2020; Schuh et al., 2017). Indeed, the current ongoing clinical trial of using azacitidine and combination chemotherapy to treat infants who have acute lymphoblastic leukemia and rearrangements in the methyltransferase-encoding gene *KMT2A* (NCT02828358; https://clinicaltrials.gov/ct2/show/NCT02828358). An additional clinical trial is investigating the use of azacytidine in combination with other drugs for AML (NCT01861002; https://clinicaltrials.gov/ct2/show/NCT01861002).

TEs have emerged as new players in multiple physiological and pathological phenomena within and beyond the hematopoietic system. Further studies that increase our understanding of the role of TEs in different systems will substantially contribute to our knowledge of the biology behind normal physiology and pathogenesis and may also reveal novel therapeutics.
